# Novel Compound Heterozygous Mutations in the *TRAPPC9* Gene in Two Siblings With Autism and Intellectual Disability

**DOI:** 10.3389/fgene.2019.00061

**Published:** 2019-02-11

**Authors:** Areerat Hnoonual, Potchanapond Graidist, Supika Kritsaneepaiboon, Pornprot Limprasert

**Affiliations:** ^1^Division of Human Genetics, Department of Pathology, Faculty of Medicine, Prince of Songkla University, Songkhla, Thailand; ^2^Department of Biomedical Sciences, Faculty of Medicine, Prince of Songkla University, Songkhla, Thailand; ^3^The Excellent Research Laboratory of Cancer Molecular Biology, Prince of Songkla University, Songkhla, Thailand; ^4^Department of Radiology, Faculty of Medicine, Prince of Songkla University, Songkhla, Thailand; ^5^Faculty of Medicine, King Mongkut’s Institute of Technology Ladkrabang, Bangkok, Thailand

**Keywords:** autism, ASD, intellectual disability, whole exome sequencing, *TRAPPC9*

## Abstract

Autism spectrum disorder (ASD) is a highly heterogeneous neurodevelopmental disorder with many contributing risk genes and loci. To date, several intellectual disability (ID) susceptibility genes have frequently been identified in ASD. Here, whole exome sequencing was carried out on a proband with ASD and identified compound heterozygous mutations of the *TRAPPC9*, which plays a role in the neuronal NF-κB signaling pathway. These mutations consisted of a novel frameshift mutation (c.2415_2416insC, p.His806Profs^∗^9) and a rare splice site mutation (c.3349+1G>A) that were segregated from an unaffected father and unaffected mother, respectively. These two heterozygous mutations were also identified in the patient’s older brother with ID. Quantitative RT-PCR revealed a significant reduction of *TRAPPC9* transcript in two siblings. This study first describes compound heterozygous mutations of the *TRAPPC9* gene in two siblings with ASD and ID, which is notable as only homozygous mutations or compound heterozygous for copy number variations and rare variant in this gene have been reported to date and associated with autosomal recessive intellectual disability. The two siblings carrying compound heterozygous *TRAPPC9* mutations presented with ID, developmental delay, microcephaly and brain abnormalities similarly to the clinical features found in almost cases with homozygous *TRAPPC9* mutation in previous studies. Together this study provides evidence that clinical manifestations of *TRAPPC9* mutations as seen in our patients with ID and autism may be broader than previous case reports have indicated.

## Introduction

Autism spectrum disorder (ASD) is a highly heterogeneous neurodevelopmental disorder characterized by deficits in social communication and social interaction, and repetitive behavior and interests. However, the major genetic causes of ASD are still largely unknown. Approximately 70% of individuals with ASD are comorbid with intellectually disability (ID) (Yeargin-Allsopp et al., 2003; Chakrabarti and Fombonne, 2005; Christensen et al., 2016). Several studies have provided evidence that genes identified for ID, especially genes involved in neuronal pathways and brain development and functions, have frequently been implicated in ASD, supporting a shared similar genetic etiology between these disorders.

To date many genes have been associated with non-syndromic ID, including *TRAPPC9* (MIM 611966) which plays a role in the neuronal NF-κB signaling pathway. Homozygous mutations, homozygous deletion, and compound heterozygous for copy number variations (CNVs)/rare variants in the *TRAPPC9* gene have been implicated with significant contributions to autosomal recessive mental retardation 13 (MRT13, MIM 613192). Most previous reported cases with *TRAPPC9* mutations presented with ID/developmental delay, microcephaly and brain abnormalities (Mir et al., 2009; Mochida et al., 2009; Philippe et al., 2009; Abou Jamra et al., 2011; Kakar et al., 2012; Marangi et al., 2013; Giorgio et al., 2016; Abbasi et al., 2017; Mortreux et al., 2018). However, to date, heterozygous mutation alone or compound heterozygous mutations in the *TRAPPC9* gene have not been identified in patient with MRT13 or other associated diseases.

Recently, whole exome sequencing (WES) studies have led to the discovery of a rapidly increasing number of novel candidate genes and causative gene mutations associated with ASD and ID. Herein, we first report compound heterozygous mutations (c.2415_2416insC and c.3349+1G>A) of the *TRAPPC9* gene in two Thai siblings with ASD and ID born to healthy and non-consanguineous parents through WES. Additionally, our findings and review of literature inform a broader understanding of the clinical features of patients with *TRAPPC9* mutations.

## Clinical Report

**FIGURE 1:**
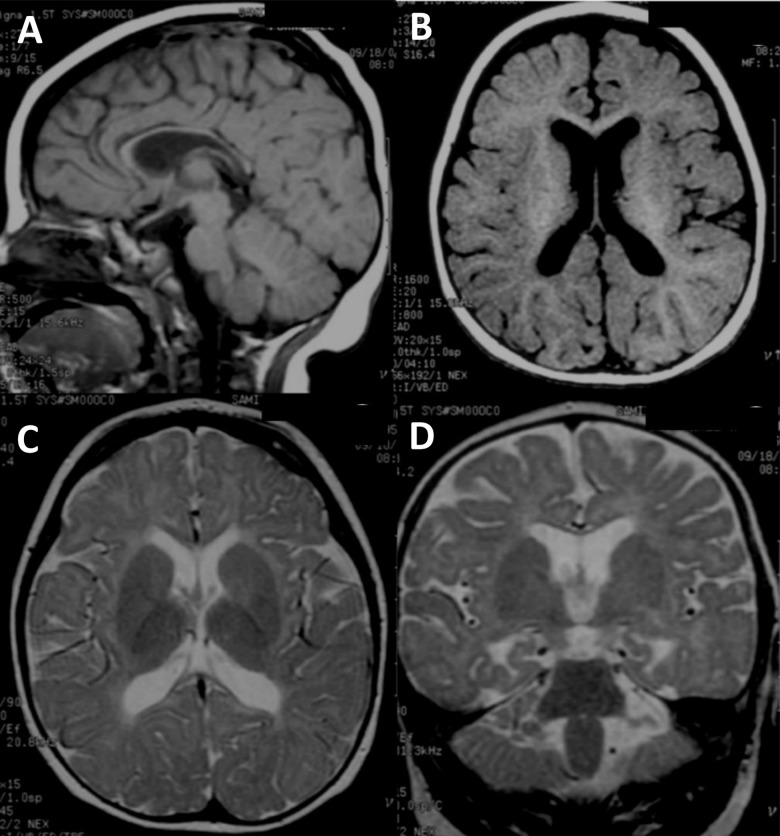
Brain MRI image of the ASD proband. **(A–D)** Brain MRI of the ASD proband at 10 months of age (II-2). **(A)** T1-weighted sagittal image shows fully formed but diffusely thin corpus callosum. The cerebellar vermis is normal. **(B)** T1-weighted axial image reveals normal gyral pattern of cerebral cortex and diminished cerebral white matter volume. **(C)** T2-weighted axial image demonstrates delayed myelination at anterior limbs of both internal capsules and diffuse white matter abnormalities seen as T2W hyperintensity at periventricular and subcortical white matter. **(D)** T2-weighted coronal image shows more obvious mild cerebral atrophy seen as enlarged CSF space and prominent temporal horns of both lateral ventricles. Diffuse white matter abnormalities at periventricular and subcortical white matter are noted.

The two affected individuals were diagnosed according to the new structured interview for Thai children with ASD for research based on the Diagnostic and Statistical Manual of Mental Disorders, Fourth Edition (DSM-IV) criteria (Hansakunachai et al., 2014). The affected proband (II:2), a 5-year-8-month female, was diagnosed with ASD from a previous study (Charalsawadi et al., 2014). She was the second child born to non-consanguineous healthy Thai parents. Her non-verbal IQ was 42 (moderate impairment) as evaluated by the Stanford-Binet Intelligence Scale : fifth edition, SB:V. Behavior and social evaluation using the Vineland Adaptive Behavior Scales (Interview edition survey form) showed that she had severe deficit in adaptive behaviors in all domains including communication, daily living skills and socialization. She started walking at the age of 24 months and to the time of our examination (5 years 8 months) had never developed speech. She presented with unilateral cleft lip at birth. The birth weight was 3,140 g (50th centile) and head circumference at birth was 34 cm (50th centile). After 3 years of age, she was found to have microcephaly (head circumference < 3rd centile). On examination, her height was 111 cm (50th centile) and weight was 24 kg (90th centile). Neither parent had a history of seizure and there was no family history of developmental delay. A brain MRI from 10 months of age is shown in Figure 1A–D. Standard karyotyping, CGG repeats of the *FMR1* and *MECP2* testings were all normal.

**FIGURE 2:**
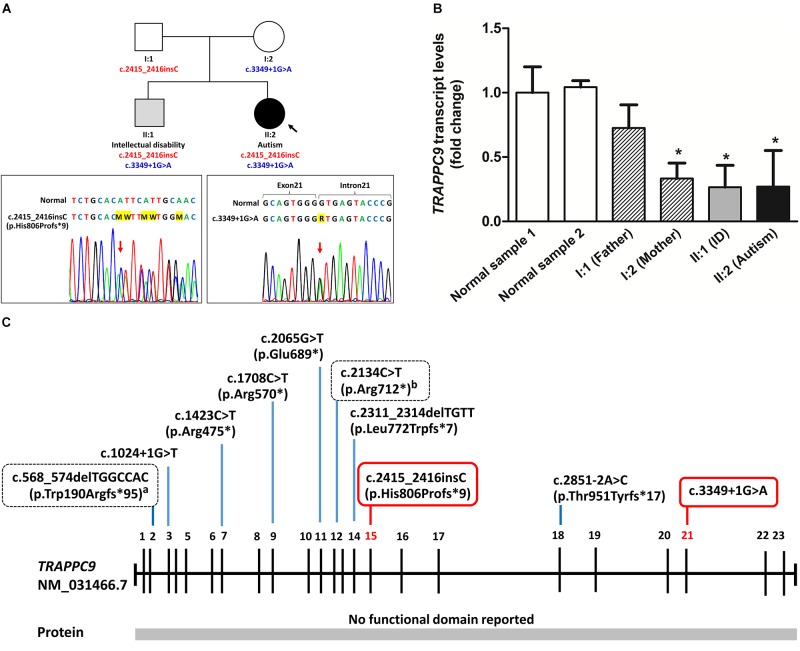
Family pedigree and molecular analysis of *TRAPPC9* mutations. **(A)** Family pedigree and sequencing of splice site mutation (c.3349+1G>A) and insertion mutation (c.2415_2416insC, p.His806Profs^∗^9) of the *TRAPPC9* gene. Unaffected individuals are shown in white. **(B)** Quantitative RT-PCR of *TRAPPC9* mRNA expression representing fold change value calculated using the ΔΔCT method for a gene expression in family members with *TRAPPC9* mutations compared to a normal control sample 1. Values are presented as the mean ± standard deviation (*n* = 3 independent experiments). ^∗^*P* < 0.05 as compared to the normal sample. **(C)** Summary of reported *TRAPPC9* mutations. All mutations have previously been reported in homozygous changes except for the two heterozygous changes which are presented in box with dashed lines. Heterozygous mutations described in this study are presented in box with solid line. ^a^Patient carries compound heterozygous for 119 kb duplication of 8q24.3 and a deletion variant (c.568_574delTGGCCAC, p.Trp190Argfs^∗^95); ^b^Patient carries compound heterozygous for 189 kb deletion of 8q24.3 and a nonsense variant (c.2134C>T, p.Arg712^∗^).

The patient’s older brother (II:1), 11-year-5-month, was moderate ID (non-verbal IQ = 42, SB:V). Results from the Vineland Adaptive Behavior Scale of this patient also showed severe deficits in all adaptive behaviors domains. He started walking at 24 months and was able to speak one word at 36 months. His birth weight was 3,280 g (50th centile). At 11 years and 5 months of age, his height was 150 cm (75th centile) and his weight was 66 kg (>97th centile). He has also no history of seizure. His face presented repaired cleft lip and cupped ears. Birth head circumference was not available, but at 1 month was 35 cm (3rd centile). A brain MRI at 9 months of age showed a thin corpus callosum, delayed myelination and reduction in cerebral white matter. The gyral pattern of the cerebral cortex was also normal (figure not shown). Standard karyotyping and CGG repeats of the *FMR1* studies were all normal. The pedigree for this family is shown in Figure 2.

We obtained written informed consent for genomic analysis of the patient and his family members and the parents provided written informed consent for the publication of the patient’s identifiable information. The study was approved by the Institutional Ethics Committee of the Faculty of Medicine, Prince of Songkla University (REC 48/364-006-3).

## Materials and Methods

### Single Nucleotide Polymorphism (SNP) Array

Single nucleotide polymorphism (SNP) array analysis was performed to identify clinically significant copy number variations (CNVs) in the proband with ASD (II:2) using the HumanCytoSNP-12 DNA Analysis BeadChip v2.1 kit (Illumina, San Diego, CA, United States), according to the manufacturer’s instructions. The data were then analyzed using BlueFuse Multi software v4.3 and GenomeStudio Data Analysis Software v. 2011.1 based on the reference human genome (hg19/GRCh37).

### Whole Exome Sequencing (WES)

Whole exome sequencing was performed on the proband with ASD (II:2) using the SureSelect Human All Exon V4 Kit (Agilent Technologies, Santa Clara, CA, United States) and sequenced on an HiSeq2000 (Illumina) with 101-bp paired-end reads. Exome sequencing data processing, base calling and primary data analysis were performed using the Illumina Real-Time Analysis (RTA) version 1.12.4 and Illumina’s CASAVA pipeline 1.8.2 with default parameters. The paired-end reads were aligned to the reference human genome (hg19/GRCh37) using the Burrows-Wheeler Aligner (BWA). Variant calling was performed using SAMtools (Li et al., 2009) and sequence variants were annotated using ANNOVAR (Wang et al., 2010). The remaining variants were then filtered against our candidate ASD gene set (762 genes) from AutDB, SFARI, and TruSight Autism genes. Subsequently, to search more widely for the cause of disease in the patient, the variants were also filtered against a candidate ID gene set (1,912 genes) from literature reviews. Sanger sequencing was then performed to confirm sequence variants and to assess segregation in the family. The details and simplified diagram of WES analysis and variants filtering are shown in Supplementary Figure S1.

### Quantitative Reverse Transcriptase Polymerase Chain Reaction (qRT-PCR)

To investigate the impact of the mutations on the transcript level of *TRAPPC9*, we performed a quantitative RT-PCR (qRT-PCR) on samples of proband and all available family members using SYBR Green Supermix (Bio-Rad) on a CFX96 Real-Time PCR detection system (Bio-Rad). Total RNA was extracted from fresh EDTA blood using TRIzol LS reagent (Invitrogen, Carlsbad, CA, United States) and cDNA was synthesized using the SuperScript^TM^ IV First-Strand cDNA synthesis system (Invitrogen) according to the manufacturer’s protocol. The primers used for the qRT-PCR have been described in a previous study (Mir et al., 2009; Supplementary Table S1). The relative quantification was calculated using a comparative CT method (ΔΔCT) and *HPRT1* (Hypoxanthine phosphoribosyltransferase 1, MIM 308000) was used as the internal control housekeeping gene. The mRNA expression for the normal control sample 1 is set a value of 1.0, and mRNA levels of all family members are determined relative to this number.

## Results

Single nucleotide polymorphism (SNP) array analysis was first performed on the proband with ASD (II:2), but no pathogenic copy number variations (CNVs) were detected. WES was then performed, which led to the identification of two heterozygous mutations of the *TRAPPC9* gene. One mutation was an insertion (NM_031466.7: c.2415_2416insC) of exon 15, leading to a frameshift and premature stop codon (p.His806Profs^∗^9). This frameshift insertion mutation was segregated from the unaffected father. In addition, this frameshift mutation was absent from the 195 unrelated Thais from our in-house exome sequencing database as well as not being found in the public databases (the 1000 Genomes Project, the NHLBI-ESP 6500 exome, the ExAC database, dbSNP 137). The second mutation was a single donor splice site mutation (NM_031466.7: c.3349+1G>A,) after exon 21, which was segregated from the unaffected mother. This splice site mutation has been reported in one case of East Asian population in the 1000 Genomes Project (minor allele frequency = 0.0002) (rs539016732). Furthermore, these two heterozygous mutations in the *TRAPPC9* gene were also identified using Sanger sequencing in the proband’s older brother with moderated ID (II:1, Figure 2A).

Quantitative RT-PCR results showed that the mRNA expression of *TRAPPC9* was slightly decreased in the father (I:1) with the frameshift mutation (Fold Change = 0.725, *P* = 0.373), but the mRNA levels of *TRAPPC9* were significantly decreased in the mother (I:2) with a rare splice site mutation (Fold Change = 0.333, *P* = 0.017), the older brother (II:1) (Fold Change = 0.266, *P* = 0.014) and the proband (II:2) (Fold Change = 0.271, *P* = 0.016) with compound heterozygous mutations compared to a normal control sample (normal sample 1) (Figure 2B). These results indicate that a splice site *TRAPPC9* mutation clearly can affect transcription of the *TRAPPC9* gene. RT-PCR followed by cDNA sequencing was then performed to investigate the consequences of the c.3349+1G>A splice site mutation on *TRAPPC9* mRNA splicing. However, we did not observe aberrant form of the *TRAPPC9* transcript in the proband, her brother and mother carrying a heterozygous splice site *TRAPPC9* mutation. These results suggest that aberrant mRNA transcripts from a splice site *TRAPPC9* mutation may sometimes be eliminated by nonsense-mediated mRNA decay (NMD) as discussed in previous studies (Mir et al., 2009; Philippe et al., 2009). However, further studies of the functional consequences of these two *TRAPPC9* mutations are needed to elucidate more definitive conclusions.

## Discussion

Trafficking Protein Particle Complex 9 (*TRAPPC9*, MIM 611966), also known as NIBP (NIK- and IKK-beta binding protein), contains 23 exons and encodes the NIBP protein which is mainly expressed in muscle, kidney, heart, placenta, and brain tissues, including postmitotic neurons of the cerebral cortex, hippocampus, and deep gray matter (Hu et al., 2005; Mochida et al., 2009). However, the function of the NIBP protein domains has not yet been determined. NIBP interacts directly with NIK and IKK-beta is involved in both the classical and alternative NF-κB signaling pathways (Hu et al., 2005) which may in turn be involved in several neuronal processes, including neuronal cells differentiation, synaptic plasticity and neurogenesis (Denis-Donini et al., 2008). Moreover, NF-κB signaling is also required for myelination in the central nervous system (Philippe et al., 2013; Blank and Prinz, 2014). A previous study found that patients with impaired NF-κB signaling presented with delayed myelination of the white matter (Philippe et al., 2013). These data support the possibility that *TRAPPC9* mutations may lead to clinical manifestations in patients with ASD and ID through impairing neuronal NF-κB signaling.

To our knowledge, six homozygous mutations in the *TRAPPC9* gene have been reported in patients with MRT13 in consanguineous families (Mir et al., 2009; Mochida et al., 2009; Philippe et al., 2009; Abou Jamra et al., 2011; Kakar et al., 2012; Marangi et al., 2013; Giorgio et al., 2016; Abbasi et al., 2017; Mortreux et al., 2018; Figure 2C and Supplementary Table S2). A nonsense *TRAPPC9* mutation (c.1423C>T, p.Arg475^∗^) has been reported in families from four different ethnicities (Israeli Arab, Syrian, Pakistani and Egyptian) (Mir et al., 2009; Mochida et al., 2009; Abou Jamra et al., 2011; Giorgio et al., 2016; Abbasi et al., 2017). The other mutations reported to be found in the *TRAPPC9* gene were nonsense mutation (c.1708C>T, p.Arg570^∗^) in a Tunisian family, nonsense mutation (c.2065G>T, p.Glu689^∗^) in a Pakistani family and a 4-bp frameshift deletion (c.2311_2314delTGTT, p.Leu772Trpfs^∗^7) in an Iranian family. In addition, splice site mutations (c.1024+1G>T and c.2851-2A>C) resulting in exon skipping with frameshift and premature truncation have been reported in a Pakistani and an Italian family, respectively. Recently, CNVs (deletion/duplication) on 8q24.3 encompassing the *TRAPPC9* gene have been reported in patients with ID (Koifman et al., 2010; Mortreux et al., 2018) in two families. Compound heterozygous CNVs/rare variants in the *TRAPPC9* gene including compound heterozygous for 119 kb duplication and a deletion variant (c.568_574delTGGCCAC, p.Trp190Argfs^∗^95), and compound heterozygous for 189 kb deletion and a nonsense variant (c.2134C>T, p.Arg712^∗^) have also been reported in patients with ID of non-consanguineous parents (Mortreux et al., 2018).

Reported clinical phenotypes associated with *TRAPPC9* mutations include dysmorphic facial features, moderate to severe ID, developmental delay, microcephaly, obesity and brain MRI abnormalities, which match the clinical features of the patients in this study carrying compound heterozygous mutations of *TRAPPC9* (Table 1). Of note, the clinical features including ID, developmental delay, microcephaly and brain abnormalities are observed in almost reported cases with *TRAPPC9* mutations. Interestingly, all known patients with *TRAPPC9* mutations from previous studies, including our patients, presented with brain abnormalities (thin corpus callosum, cerebral and cerebellar hypoplasia), supporting the important role of *TRAPPC9* in brain development and functions. Patients in this study also show autistic feature and cleft lip which have been reported in very rare cases with *TRAPPC9* mutations, suggesting a broader spectrum of clinical manifestations of *TRAPPC9* mutations as seen in our patients, compared to previous case reports.

**Table 1:** Comparison of available clinical features between previous case reports with *TRAPPC9* mutations (41 patients/15 families) and patients in this study.

Clinical features	Previous reports	Present study		Total N
		Patient II:1	Patient II:2	
Origin	Information in Supplementary Table S2	Thai		–
No. affected individuals	41	2		43
Male:Female	16:25	1:1		17:26
No. family	15	1		16
Diagnosis	All ID	ID	ASD	–
*TRAPPC9* mutation	Homozygous mutations, homozygous deletion/duplication, compound heterozygous CNV + rare variant (Supplementary Table S2)	Compound heterozygous mutations c.2415_2416insC (p.His806Profs^∗^9) and c.3349+1G>A		–
Developmental delay	41/41	Yes	Yes	43/43 (100%)
Autistic features	3/17	No	Yes	4/19 (21.1%)
Microcephaly	37/39	Yes	Yes	39/41 (95.1)
Obesity	10/21	Yes	Yes	12/23 (52.2%)
Seizure	5/29	No	No	5/31 (16.1%)
Hand-flapping movements	8/10	No	Yes	9/12 (75%)
Frequent sleep awakenings	3/3	Yes	No	4/5 (80%)
**Brain abnormalities**				
Thin corpus callosum	15/15	Yes	Yes	17/17 (100%)
Cerebral hypoplasia	9/9	Yes	Yes	11/11 (100%)
Cerebellar hypoplasia	7/8	No	No	7/10 (70%)
Abnormal signal of white matter	15/16	Yes	Yes	17/18 (94.4%)
Delayed myelination	2/3	Yes	Yes	4/5 (80%)
**Dysmorphic facial features**^a^	19/34	Yes	Yes	21/36 (58.3%)
Brachycephaly	4^a^	Yes	Yes	6
Round face	4^a^	Yes	Yes	6
Wide nasal bridge	7^a^	No	No	7
Synophrys	10^a^	No	No	10
Hypertelorism	4^a^	No	No	4
Deep-set-eyes	5^a^	No	No	5
Short philtrum	8^a^	No	No	8
Thin upper lip	5^a^	No	No	5
Cleft lip	1/41^b^	Yes	Yes	3/43 (7.0%)


*ID, intellectual disability; ASD, autism spectrum disorder; CNV, copy number variation. ^a^Not complete report, ^b^assumption of the authors. Percentage may not reflect to the real figure because it depend on available report.*

## Concluding Remarks

This study is the first to report the identification of novel compound heterozygous mutations of the *TRAPPC9* gene in two Thai siblings with ASD and ID. The combination of these two *TRAPPC9* mutations are most likely the cause of clinical features in the patients, especially ID, developmental delay, microcephaly and brain abnormalities. We identify the first case diagnosed with ASD carrying *TRAPPC9* mutations. Thus, our findings suggest that *TRAPPC9* mutations may constitute one of the genetic risk factors for ASD in the patient.

## Author Contributions

PL and AH designed the study and wrote the manuscript. PL collected the patients’ clinical information. SK interpreted the brain MRIs of the patients. AH performed the analysis of WES data. AH and PG performed the molecular analysis and interpreted the results. All authors reviewed and approved the final manuscript.

## Conflict of Interest Statement

The authors declare that the research was conducted in the absence of any commercial or financial relationships that could be construed as a potential conflict of interest.
