# Supplementary material for: Novel Compound Heterozygous Mutations in the TRAPPC9 Gene in Two Siblings With Autism and Intellectual Disability
Source: Front Genet. 2019 Feb 11;10:61. doi: 10.3389/fgene.2019.00061 (PMC6396715; doi:10.3389/fgene.2019.00061)
Supplement: Supplementary file 1 [file Data_Sheet_1.pdf]

Supplementary Material

Novel compound heterozygous mutations in the *TRAPPC9* gene in two siblings with autism and intellectual disability

Areerat Hnoonual, Potchanapond Graidist, Supika Kritsaneepaiboon, Pornprot Limprasert*

*** Correspondence:** Pornprot Limprasert: pornprot.li@kmitl.ac.th

Materials and Methods

Filtering steps of the whole exome sequencing (WES)

In the variant filtering steps of WES analysis, we first excluded all variants with a base quality score < 20. The variants with a high probability of affecting gene functions including nonsense, frameshift, splice site, indel and missense variations were included. In the next step, variants located on conserved genomic regions based on 46-way alignment were included. We then excluded all the sequence variants reported in dbSNP 137, the 1000 Genomes Project, Exome Variant Server, Exome Aggregation Consortium and our in-house exome sequencing data from 195 unrelated Thai individuals with minor allele frequency (MAF) > 0.01 to identify novel and rare variants. Finally, we chose to filter down the list of potentially causative variants in this patient based mainly on the known 762 ASD candidate genes from AutDB, SFARI and TruSight Autism genes and 1,912 intellectual disability (ID) genes from databases and literature reviews. Several in *silico* prediction tools including SIFT, Polyphen2, Mutation Taster, and FATHMM software programs were used to predict the effect of identified variants on protein function, stability and structure. Interpretation of potentially causative variants was performed based on guidelines of the American College of Medical Genetics and Genomics (ACMG) (Richards et al., 2015). The simplified diagram of WES analysis is shown in Supplementary Figure S1.

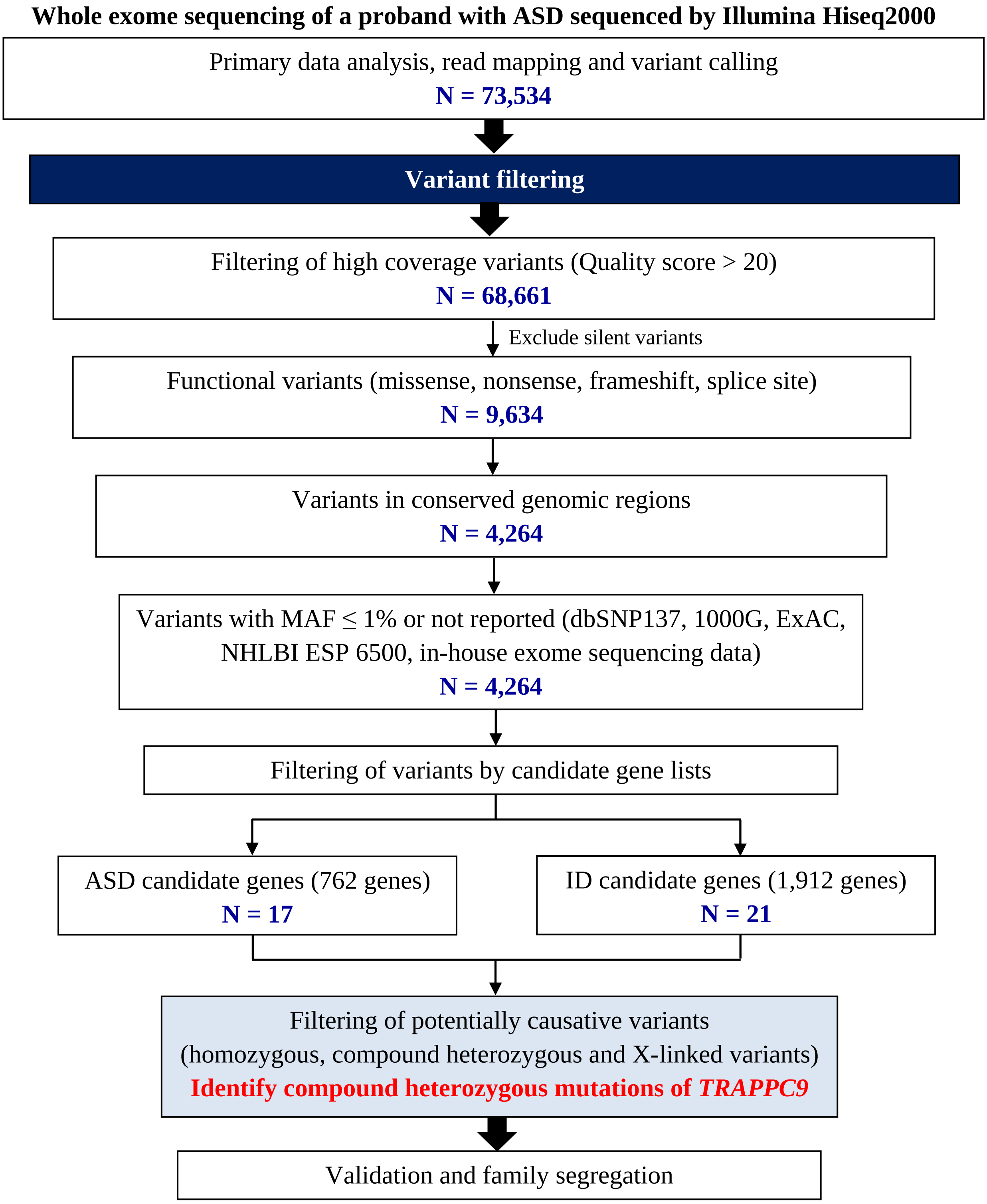

Supplementary Figure S1. Variant filtering strategy following whole exome sequencing of a proband with ASD. Compound heterozygous mutations of the *TRAPPC9* gene, a novel frameshift mutation (c.2415_2416insC, p.His806Profs*9) and a rare splice site mutation (c.3349+1G>A), were identified. Abbreviations: ASD, autism spectrum disorder; ID, intellectual disability; Indels; insertions and deletions; 1000G, 1000 Genomes Project; ESP 6500, Exome Variant Server (NHLBI Exome Sequencing Project (ESP) 6500 exome); ExAC, Exome Aggregation Consortium; MAF, minor allele frequency; N, number of remaining variants

Result

Whole exome sequencing

Whole exome sequencing was performed on an ASD patient with 80% coverage across the target regions and 113.1X average sequence read depth, with 97% covered with at least 10X coverage. There was a total number of 73,534 variants including 67,394 single nucleotide variants (SNVs) and 6,140 indels. Of these, 20,075 SNVs and 494 indels were located on protein-coding regions.

Bioinformatic analysis to narrow down candidate variants resulted in a total number of 17 variants in ASD candidate genes and 25 variants in ID candidate genes. Among these, mutations in only *TRAPPC9* gene were identified in the compound heterozygous state, and were selected for further validation and discussion based on supportive evidence in the literature reviews.

Supplementary Table S1 Primers used in this study**.**

Gene	Exon	Forward primer sequence (5'->3')	Reverse primer sequence (5'->3')	Product Size
**Primers used to confirm whole exome sequencing results**				
TRAPPC9 (c.2415_2416insC)	Exon 15	TGGTGATTCTTCTTTGGGAAG	CTGACTTCAACTGAATCCACAAA	374 bp
TRAPPC9 (c.3349+1G>A)	Exon 21	CCCATCTGAGGGTCTCTGTC	TTCCCGTGATGACCTTCAGT	309 bp
**Primers used for quantitative RT-PCR (Mir et al., 2009)**				
TRAPPC9 (Target gene)	Spans Exon 2 and 3	GATAAGATCCCCCTTCTCTGTGTC	CTTGGCACCGCTTCTTGTAAT	91 bp
HPRT (Reference gene)	Spans Exons 6-8	TGGTCAGGCAGTATAATCCAAA	TCAAGGGCATATCCTACAACAA	136 bp

Supplementary Table S2 Clinical features of patients with *TRAPPC9* mutations from the literature review.

**Clinical features**	**Homozygous mutations, Homozygous deletion/duplication**													**Compound heterozygous CNV + rare variant**		**Total previous case reports**
	**Mir et al., 2009**		**Mochida et al., 2009**	**Philippe et al., 2009**	**Koifman et al., 2010**	**Abou Jamra et al., 2011**	**Kakar et al., 2012**	**Marangi et al., 2013**	**Giorgio et al., 2016**	**Abbasi et al., 2017**		**Mortreux et al., 2018**				
Origin	Pakistan	Iranian	Israeli Arab	Tunisian	Filipino	Syrian	Pakistan	Italian	Egyptian	Pakistan	Pakistan	Algerian	Tunisian	Italian	French	
Consanguinity	Yes	Yes	Yes	Yes	Yes	Yes	Yes	Yes	Yes	Yes	Yes	Yes	Yes	No	No	
No. Affected individuals	7	3	3	3	1	6	3	2	1	3	3	1	3	1	1	**41**
Male: Female	1:6	3:0	0:3	3:0	0:1	3:3	0:3	0:2	0:1	2:1	3:0	0:1	1:2	0:1	0:1	**16:25**
Diagnosis	ID	ID	ID	ID	ID	ID	ID	ID	ID	ID	ID	ID	ID	ID	ID	**ID**
TRAPPC9 mutation	c.1423C>T (p.Arg475*)	c.2311_2314 delTGTT (p.Leu772Trpfs*7)	c.1423C>T (p.Arg475*)	c.1708C>T (p.Arg570*)	141.46 kb deletion of 8q24.3 including *TRAPPC9*	c.1423C>T (p.Arg475*)	c.1024+1G>T	c.2851-2A>C (p.Thr951Tyrfs*17)	c.1423C>T (p.Arg475*)	c.2065G>T (p.Glu689*)	c.1423C>T (p.Arg475*)	115 kb duplication in *TRAPPC9*[a]	c.1708C>T (p.Arg570*)	119 kb duplication in *TRAPPC9* + deletion variant[b]	189 kb deletion in *TRAPPC9* + c.2134C>T, (p.Arg712*)[c]	
Developmental delay	7/7	3/3	3/3	3/3	1/1	6/6	3/3	2/2	1/1	3/3	3/3	1/1	3/3	1/1	1/1	**41/41 (100%)**
Autistic features	0/3	NA	0/3	NA	NA	NA	0/3	0/2	NA	NA	NA	0/1	1/3	1/1	1/1	**3/17 (17.6%)**
Microcephaly	5/6	3/3	3/3	3/3	1/1	6/6	3/3	2/2	1/1	2/2	3/3	1/1	3/3	0/1	1/1	**37/39 (94.9%)**
Obesity	NA	NA	NA	3/3	NA	NA	0/3	2/2	1/1	0/3	0/3	0/1	3/3	1/1	0/1	**10/21 (47.6%)**
Seizure	1/3	0/3	NA	NA	NA	1/6	0/3	1/2	NA	1/3	1/3	0/1	0/3	0/1	0/1	**5/29 (17.2%)**
Hand-flapping movements	NA	NA	1/3	NA	NA	6/6	NA	NA	1/1	NA	NA	NA	NA	NA	NA	**8/10 (80%)**
Frequent sleep awakenings	NA	NA	NA	NA	NA	NA	NA	2/2	1/1	NA	NA	NA	NA	NA	NA	**3/3 (100%)**
**Brain abnormalities**																
Thin corpus callosum	3/3	NA	2/2	NA	1/1	NA	NA	2/2	1/1	NA	NA	1/1	3/3	1/1	1/1	**15/15 (100%)**

Cerebral hypoplasia	3/3	NA	2/2	NA	1/1	NA	NA	2/2	1/1	NA	NA	NA	NA	NA	NA	**9/9 (100%)**
Cerebellar hypoplasia	3/3	NA	1/1	NA	1/1	NA	NA	2/2	0/1	NA	NA	NA	NA	NA	NA	**7/8 (87.5%)**
Abnormal signal of white matter	3/3	NA	0/1	2/2	1/1	NA	NA	2/2	1/1	NA	NA	1/1	3/3	1/1	1/1	**15/16 (93.75%)**
Delayed myelination	NA	NA	NA	0/1	1/1	NA	NA	NA	1/1	NA	NA	NA	NA	NA	NA	**2/3 (66.7%)**
**Dysmorphic facial features[d]**	0/3	0/3	NA	2/3	1/1	6/6	0/3	2/2	1/1	1/3	0/3	1/1	3/3	1/1	1/1	**19/34 (55.9%)**
Brachycephaly	NA	NA	NA	NA	1	NA	NA	2	1	NA	NA	NA	NA	NA	NA	**4**
Round face	NA	NA	NA	NA	1	NA	NA	2	1	NA	NA	NA	NA	NA	NA	**4**
Wide nasal bridge	NA	NA	NA	NA	1	NA	NA	2	1	NA	NA	0	2	1	0	**7**
Synophrys	NA	NA	NA	NA	1	6	NA	2	1	NA	NA	NA	NA	NA	NA	**10**
Hypertelorism	NA	NA	NA	2	NA	NA	NA	2	NA	NA	NA	NA	NA	NA	NA	**4**
Deep-set-eyes	NA	NA	NA	NA	1	NA	3	NA	1	NA	NA	NA	NA	NA	NA	**5**
Short philtrum	NA	NA	NA	2	1	NA	NA	NA	NA	NA	NA	1	2	1	1	**8**
Thin upper lip	NA	NA	NA	NA	NA	NA	NA	2	1	NA	NA	0	0	1	1	**5**
Cleft lip	0/7[e]	0/3[e]	0/3[e]	1/3	0/1[e]	0/6[e]	0/3[e]	0/2[e]	0/1[e]	0/3[e]	0/3[e]	0/1[e]	0/3[e]	0/1[e]	0/1[e]	**1/41 (2.4%)**

NA, not available; ID, intellectual disability; ASD, autism spectrum disorder

[a] homozygous 115 kb duplication of 8q24.3 including out-of-frame exons 2–9 in *TRAPPC9*.

[b] compound heterozygous for 119 kb duplication of 8q24.3 including in-frame exons 9–16 in *TRAPPC9* and a deletion variant (c.568_574delTGGCCAC, p.Trp190Argfs*95).

[c] compound heterozygous for 189 kb deletion of 8q24.3 including out-of-frame exons 18 and 19 and a nonsense variant (c.2134C>T, p.Arg712*).

[d] not complete report.

[e] assumption of the authors.

References

Abbasi, A.A., Blaesius, K., Hu, H., Latif, Z., Picker-Minh, S., Khan, M.N., et al. (2017). Identification of a novel homozygous TRAPPC9 gene mutation causing non-syndromic intellectual disability, speech disorder, and secondary microcephaly. *Am J Med Genet B Neuropsychiatr Genet.* 174(8)**,** 839-845. doi: 10.1002/ajmg.b.32602.

Abou Jamra, R., Wohlfart, S., Zweier, M., Uebe, S., Priebe, L., Ekici, A., et al. (2011). Homozygosity mapping in 64 Syrian consanguineous families with non-specific intellectual disability reveals 11 novel loci and high heterogeneity. *Eur J Hum Genet.* 19(11)**,** 1161-1166. doi: 10.1038/ejhg.2011.98.

Giorgio, E., Ciolfi, A., Biamino, E., Caputo, V., Di Gregorio, E., Belligni, E.F., et al. (2016). Whole exome sequencing is necessary to clarify ID/DD cases with de novo copy number variants of uncertain significance: Two proof-of-concept examples. *Am J Med Genet A.* 170(7)**,** 1772-1779. doi: 10.1002/ajmg.a.37649.

Kakar, N., Goebel, I., Daud, S., Nurnberg, G., Agha, N., Ahmad, A., et al. (2012). A homozygous splice site mutation in TRAPPC9 causes intellectual disability and microcephaly. *Eur J Med Genet.* 55(12)**,** 727-731. doi: 10.1016/j.ejmg.2012.08.010.

Koifman, A., Feigenbaum, A., Bi, W., Shaffer, L.G., Rosenfeld, J., Blaser, S., et al. (2010). A homozygous deletion of 8q24.3 including the NIBP gene associated with severe developmental delay, dysgenesis of the corpus callosum, and dysmorphic facial features. *Am J Med Genet A.* 152a(5)**,** 1268-1272. doi: 10.1002/ajmg.a.33319.

Marangi, G., Leuzzi, V., Manti, F., Lattante, S., Orteschi, D., Pecile, V., et al. (2013). TRAPPC9-related autosomal recessive intellectual disability: report of a new mutation and clinical phenotype. *Eur J Hum Genet.* 21(2)**,** 229-232. doi: 10.1038/ejhg.2012.79.

Mir, A., Kaufman, L., Noor, A., Motazacker, M.M., Jamil, T., Azam, M., et al. (2009). Identification of mutations in TRAPPC9, which encodes the NIK- and IKK-beta-binding protein, in nonsyndromic autosomal-recessive mental retardation. *Am J Hum Genet.* 85(6)**,** 909-915. doi: 10.1016/j.ajhg.2009.11.009.

Mochida, G.H., Mahajnah, M., Hill, A.D., Basel-Vanagaite, L., Gleason, D., Hill, R.S., et al. (2009). A truncating mutation of TRAPPC9 is associated with autosomal-recessive intellectual disability and postnatal microcephaly. *Am J Hum Genet.* 85(6)**,** 897-902. doi: 10.1016/j.ajhg.2009.10.027.

Mortreux, J., Busa, T., Germain, D.P., Nadeau, G., Puechberty, J., Coubes, C., et al. (2018). The role of CNVs in the etiology of rare autosomal recessive disorders: the example of TRAPPC9-associated intellectual disability. *Eur J Hum Genet.* 26(1)**,** 143-148. doi: 10.1038/s41431-017-0018-x.

Philippe, O., Rio, M., Carioux, A., Plaza, J.M., Guigue, P., Molinari, F., et al. (2009). Combination of linkage mapping and microarray-expression analysis identifies NF-kappaB signaling defect as a cause of autosomal-recessive mental retardation. *Am J Hum Genet.* 85(6)**,** 903-908. doi: 10.1016/j.ajhg.2009.11.007.

Richards, S., Aziz, N., Bale, S., Bick, D., Das, S., Gastier-Foster, J., et al. (2015). Standards and guidelines for the interpretation of sequence variants: a joint consensus recommendation of the American College of Medical Genetics and Genomics and the Association for Molecular Pathology. *Genet Med* 17(5), 405-424. doi:10.1038/gim.2015.30.
